# Long-Lived Hole Accumulation in Al:SrTiO_3_/Rh–Cr Photocatalyst Systems under Continuous Irradiation and Its Correlation with Overall Water Splitting Efficiency

**DOI:** 10.1021/jacs.5c07521

**Published:** 2025-09-11

**Authors:** Anna A. Wilson, Benjamin Moss, Aysha A. Riaz, Curran Kalha, Pardeep K. Thakur, Tien-Lin Lee, Anna Regoutz, Tsuyoshi Takata, Takashi Hisatomi, Kazunari Domen, James R. Durrant

**Affiliations:** † Department of Chemistry, Centre for Processable Electronics, 4615 Imperial College London, London SW7 2AZ, United Kingdom; ‡ Resnik Centre for Sustainability, California Institute of Technology, Los Angeles, California 91125, United States; § Department of Chemistry, 4919 University College London, 20 Gordon Street, London WC1E 7HU, United Kingdom; ∥ Diamond Light Source Ltd., Diamond House, Harwell Science and Innovation Campus, Didcot OX11 0DE, United Kingdom; ⊥ Department of Chemistry, University of Oxford University, Inorganic Chemistry Laboratory, South Parks Road, Oxford OX1 3QR, United Kingdom; # Research Initiative for Supra-Materials, Shinshu University, 4-17-1 Wakasato, Nagano-shi, Nagano 380-0928, Japan; ¶ Institute for Aqua Regeneration, Research Initiative for Supra-Materials, Shinshu University, 4-17-1 Wakasato, Nagano-shi, Nagano 380-0928, Japan; h Office of University Professors, The University of Tokyo, 2-11-16 Yayoi, Bunkyo-ku, Tokyo 113-8656, Japan; i Department of Materials Science and Engineering, Swansea University, Swansea SA2 8PP, United Kingdom

## Abstract

Photocatalytic water splitting offers a scalable and potentially low-cost route for the production of renewable hydrogen. Recently, a state-of-the-art system based on flux-mediated Al^3+^-doped SrTiO_3_, modified with Rh–Cr-based proton reduction and CoOOH water oxidation cocatalysts, achieved apparent quantum yields for unassisted water splitting of up to 93%. Herein, we focus on the role of Al^3+^ doping and Rh–Cr-based cocatalyst deposition on the accumulation and reaction dynamics of the long-lived holes required to drive water oxidation. We employ in situ and operando photoinduced absorption spectroscopy (PIAS) under water splitting conditions complemented by X-ray photoelectron spectroscopy (XPS). XPS data indicate that Al^3+^ doping suppresses surface Ti^3+^ defect states, coinciding with a 5-fold increase in the accumulation of long-lived SrTiO_3_ holes observed by PIAS. Rh–Cr-based cocatalyst addition is observed to further enhance the yield and lifetime (s–10 s time scales) of these photoaccumulated holes, assigned to the efficient electron extraction by this cocatalyst. These photoaccumulated holes exhibit fast (ca. 1 s) and slow (ca. 10 s) decay phases. While the dominant fast phase is assigned to the desired water oxidation reaction, the slow phase is assigned to deeply trapped unreactive holes; the yield of these unreactive holes is suppressed by facet-selective photodeposition of cocatalysts or preillumination. These results provide key insights into how Al:SrTiO_3_ functionalized by Rh–Cr-based cocatalysts accumulates oxidizing holes with lifetimes long enough to drive the kinetically challenging water oxidation reaction, thus achieving remarkably high quantum efficiencies for overall water splitting, insights which can be applied in the design of future photocatalytic materials.

## Graphical abstract

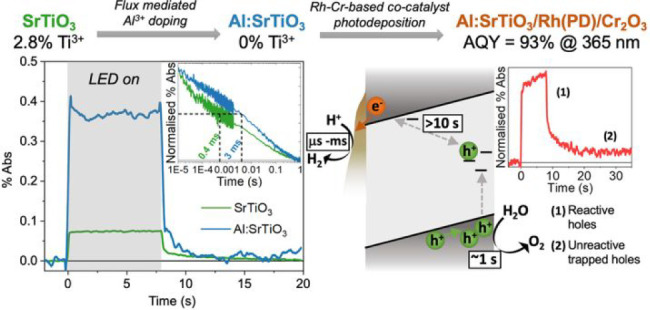

## Introduction

Photocatalytic overall water splitting offers a means of harnessing solar energy to drive the synthesis of green hydrogen. The use of particulate photocatalysts for water splitting presents notable advantages over alternative methods, including photoelectrochemical (PEC) water splitting and photovoltaic coupled electrolysis (PV-EC). These include the absence of electrical contacts or electrolytes and upscalable fabrication routes, offering the potential of large-scale hydrogen production with low operational costs.^1–3^ The scalability of particulate photocatalysts has been demonstrated through pilot projects, including one that safely and stably produces hydrogen on the 100 m^2^ scale.^4^ For photocatalytic water splitting systems to achieve their potential, they must efficiently utilize the solar spectrum to produce photogenerated charges that drive water splitting reactions with a high apparent quantum yield (AQY). To obtain a high AQY, charges must be efficiently separated such that water splitting reactions, and in particular the kinetically slow water oxidation reaction,^5,6^ outcompete recombination. This is a substantive kinetic challenge, requiring in some materials increases in carrier lifetimes by up to 9 orders of magnitude.^7,8^ In photocatalytic systems, the absence of externally applied bias (and ideally scavengers) means that charge separation and lifetime gain must be achieved solely due to the intrinsic photocatalyst properties. With this in mind, it is remarkable that a near-*unity* AQY has recently been recorded using Al^3+^ doped SrTiO_3_ modified with Cr_2_O_3_/Rh and CoOOH cocatalysts.^9^ While there has been significant progress in understanding their fast time scale dynamics,^10^ the factors determining how this system can generate reactive holes with lifetimes long enough to drive water oxidation remain poorly understood.

To obtain the photocatalyst system with near-*unity* quantum efficiency, the compositional flexibility of the SrTiO_3_ perovskite structure was exploited by its aliovalent doping with Al^3+^, aimed at suppressing Ti^3+^ states that can serve as recombination centers and limit photocatalytic performance.^11,12^ The Al^3+^ doped SrTiO_3_ (Al:SrTiO_3_) was subsequently modified with a Rh–Cr-based cocatalyst for proton reduction and suppression of back reactions.^13,14^ This cocatalyst can be deposited via impregnation (Al:SrTiO_3_/RhCrO_*x*_(IMP)) or photodeposition (Al:SrTiO_3_/Rh­(PD)/Cr_2_O_3_), to obtain the active photocatalyst compositions, noting that following photodeposition the cocatalyst is typically present as a separated phase between Rh and Cr_2_O_3_.^9,15^ In addition to this reduction cocatalyst, CoOOH is photodeposited to serve as an overlayer for stability in the case of Al:SrTiO_3_/RhCrO_*x*_(IMP) and to further improve photocatalytic activity for Al:SrTiO_3_/Rh­(PD)/Cr_2_O_3_. An AQY of 56% at 365 nm was achieved by Al:SrTiO_3_/RhCrO_*x*_(IMP),^15^ noting that AQYs exceeding 50% are rare for overall water splitting photocatalysts.^6,16,17^ Meanwhile, a near-*unity* AQY (93% at 365 nm) has been achieved by the Al:SrTiO_3_/Rh­(PD)/Cr_2_O_3_/CoOOH system employing facet-selective photodeposition of cocatalysts,^9^ suggested to be facilitated by an internal electric field between distinct oxidation and reduction facets. These systems exhibit the highest AQYs reported to date for overall photocatalytic water splitting,^4,9,15,18^ although their wide band gap (∼3.2 eV) limits utilization of visible light. Thus, determining the properties that enable their impressive AQY is key for establishing design principles that can be translated to new photocatalytic materials with greater visible light activity.

Several recent studies have addressed the charge carrier dynamics of SrTiO_3_ and its relevance to its high photocatalytic performance. ps–ns studies, including our own,^10,19,20^ have reported remarkably slow recombination kinetics in SrTiO_3_, with a bimolecular recombination rate constant at least two magnitudes slower than alternative metal oxides.^19^ We have suggested that this may be associated with an anomalously high dielectric constant of SrTiO_3_ reducing the Coulomb capture radius of photogenerated charges.^19^ Murthy et al. have demonstrated that Al^3+^ doping, and the subsequent suppression of Ti^3+^ states, further increases the bulk charge carrier lifetime and enables efficient electron extraction to a Rh cocatalyst.^10^ Our studies of SrTiO_3_ photoanodes on ms–s time scales have reported long-lived holes, even in the absence of applied bias, but also highlighted that deep charge trapping limits photoanode performance.^21^ Further studies of both SrTiO_3_ and Al:SrTiO_3_ have identified the roles of defects and Al^3+^ doping in extending charge carrier lifetimes and improving photocatalytic activity,^22–24^ in addition to highlighting the sensitivity of the charge carrier dynamics and photocatalytic activity to the source of the materials, attributed to differing morphologies and defects.^23,24^

*In situ* time-resolved techniques, such as those employed herein, offer the benefit of measuring the charge carrier dynamics relevant to photocatalyst operation. *In situ* photoinduced absorption spectroscopy (PIAS) offers the additional benefit of probing these charge carrier dynamics under quasi-continuous excitation in a manner similar to sunlight. Of particular interest given the slow kinetics of water oxidation, reported previously to be on the ms–s time scale for a range of metal oxides including SrTiO_3_,^21,25^ is the ability to measure the photoaccumulation and ms–s dynamics of long-lived hole densities under the quasi-steady-state *operando* conditions of PIAS. While transient spectroscopy and microwave conductivity measurements have been conducted previously on Al:SrTiO_3_ photocatalysts modified by Rh–Cr-based cocatalysts on ns−μs time scales,^10^ no such studies have previously been reported for these systems on the ms–s time scale critical for efficient water oxidation. Due to the sensitivity of charge carrier dynamics and photocatalytic activity to the source of the materials,^23,24^ spectroscopic investigations should be conducted on the high-performing SrTiO_3_-based photocatalysts to ensure that the findings are relevant to these materials. With this in mind, this work investigates the state-of-the-art Al:SrTiO_3_/RhCrO_*x*_(IMP) and Al:SrTiO_3_/Rh­(PD)/Cr_2_O_3_ photocatalysts synthesized as reported previously.^9,15^ Herein, we measure and explain the suppressed recombination and increased steady-state photoaccumulation of reactive holes achieved by Al^3+^ doping of SrTiO_3_. We furthermore investigate the effects of Rh–Cr-based cocatalysts on the charge carrier dynamics of Al:SrTiO_3_ and demonstrate the ability of Al:SrTiO_3_/RhCrO_*x*_(IMP) and Al:SrTiO_3_/Rh­(PD)/Cr_2_O_3_ to accommodate significantly more efficient accumulation of long-lived holes without a concurrent increase in recombination. This is attributed to the synergetic effects of Al^3+^ doping and Rh–Cr-based cocatalysts. Although long-lived (ms–s) hole accumulation is important for the slow multihole water oxidation reaction, in Al:SrTiO_3_/RhCrO_*x*_(IMP) we observe the accumulation of even longer long-lived (ca. 10 s) holes, assigned to deeply trapped, and therefore unreactive, species. Meanwhile, in the Al:SrTiO_3_/Rh­(PD)/Cr_2_O_3_-based system, we observe a strongly suppressed accumulation of these deeply trapped holes. Thus, we attribute the near-*unity* AQY of Al:SrTiO_3_/Rh­(PD)/Cr_2_O_3_/CoOOH to its capacity to efficiently separate charge while avoiding the formation of inactive trapped species.

## Results

The SrTiO_3_ studied herein is as-purchased from Wako chemicals, and the Al:SrTiO_3_ was prepared by a flux-mediated doping procedure.^12^ Following this, Rh–Cr-based cocatalysts were deposited by either impregnation or photodeposition, to yield Al:SrTiO_3_/RhCrO_*x*_(IMP) and Al:SrTiO_3_/Rh­(PD)/Cr_2_O_3_, respectively.^9,15,18^ PIAS and diffuse reflectance transient spectroscopy (DRTS) measurements were undertaken on photocatalyst sheets comprised of immobilized powders on quartz substrates, mixed with SiO_2_ nanoparticles (see SI for fabrication method) to facilitate water penetration and bubble evolution, with the setup used for these techniques illustrated in Figure S1.^15^ The effects of Al^3+^ doping and subsequent Rh–Cr-based cocatalyst deposition on physical properties were characterized by X-ray diffractometry (XRD), scanning electron microscopy (SEM), and UV–vis spectrometry, yielding similar results to those reported previously,^9,12,15,18^ complimented by soft X-ray photoelectron spectroscopy (SXPS) and hard X-ray photoelectron spectroscopy (HAXPES) as discussed in more detail below. The experimental details for the material synthesis, characterization, and time-resolved spectroscopic techniques are included in the SI.

### Characterization of the SrTiO_3_-Based Photocatalyst Materials

The XRD patterns of all compositions correspond to those expected for SrTiO_3_ (Figure S2).^26^ The full width half-maximum (fwhm) of the {100} diffraction peak decreases following Al^3+^ doping and is maintained following Rh–Cr-based cocatalyst deposition (Table S2), suggesting a decrease in crystal strain and increased crystallinity compared to unmodified SrTiO_3_.^12,24^ The SrTiO_3_ particles observed by SEM are irregular shapes with diameters of 150–500 nm (Figure 1a). The flux-mediated Al^3+^ doping yields larger particles with diameters of 200–800 nm (Figure 1b). These Al:SrTiO_3_ particles have truncated cubic structures, the equilibrium crystal shape of SrTiO_3_.^12,27^ In Al:SrTiO_3_/Rh­(PD)/Cr_2_O_3_, a distribution of smaller particles is apparent on some facets, most likely corresponding to Cr_2_O_3_/Rh particles (Figure 1d). This is consistent with previous reports of selective deposition of Cr_2_O_3_/Rh onto the {100} reduction facets by the photodeposition method, made possible by the energy difference between the oxidation and reduction facets of the truncated cubic Al:SrTiO_3_ particles.^9,28^ Minimal changes in UV–vis absorption properties are observed following Al^3+^ doping and cocatalyst deposition (Figures S3 and S4), indicating that the improvements in performance achieved by these modifications are not the result of increased light harvesting.

**Figure 1:**
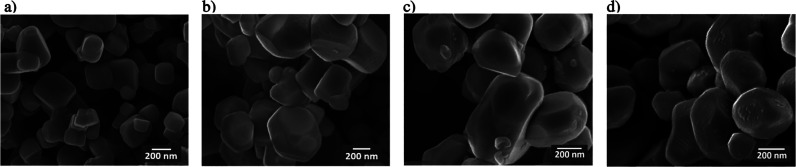
SEM images of a) SrTiO_3_, b) truncated cubic Al:SrTiO_3_ fabricated by flux-mediated Al^3+^ doping, c) Al:SrTiO_3_/RhCrO_*x*_(IMP) following unselective deposition of RhCrO_*x*_ via impregnation, and d) Al:SrTiO_3_/Rh­(PD)/Cr_2_O_3_ following selective photodeposition of the Rh–Cr-based cocatalyst onto the {100} reduction facets.

### Investigation of the Chemical Composition and Ti^3+^ Defect States

We now turn to the chemical composition and electronic structure of the materials measured by SXPS employing Al Kα irradiation in a laboratory setting. In SrTiO_3_, a small but significant shoulder is observed in the Ti 2p_3/2_ spectrum (Figure 2a), corresponding to a Ti^3+^ concentration of 1.5 rel. at. %. Al^3+^ doping introduces Al^3+^ states (Figure S5) and suppresses observable Ti^3+^ states (Figure 2a) within the resolution of the measurement, which is consistent with previous reports.^11,12,29^ These SXPS spectra also indicate that the dominant Rh oxidation state in the Rh–Cr-based cocatalysts is dependent on the deposition method, with a greater contribution of Rh metal to the Rh 3d spectra in Al:SrTiO_3_/Rh­(PD)/Cr_2_O_3_ compared to Al:SrTiO_3_/RhCrO_*x*_(IMP) (Figure S6), consistent with a greater phase segregation between Rh and CrO_*x*_ following photodeposition.

**Figure 2:**
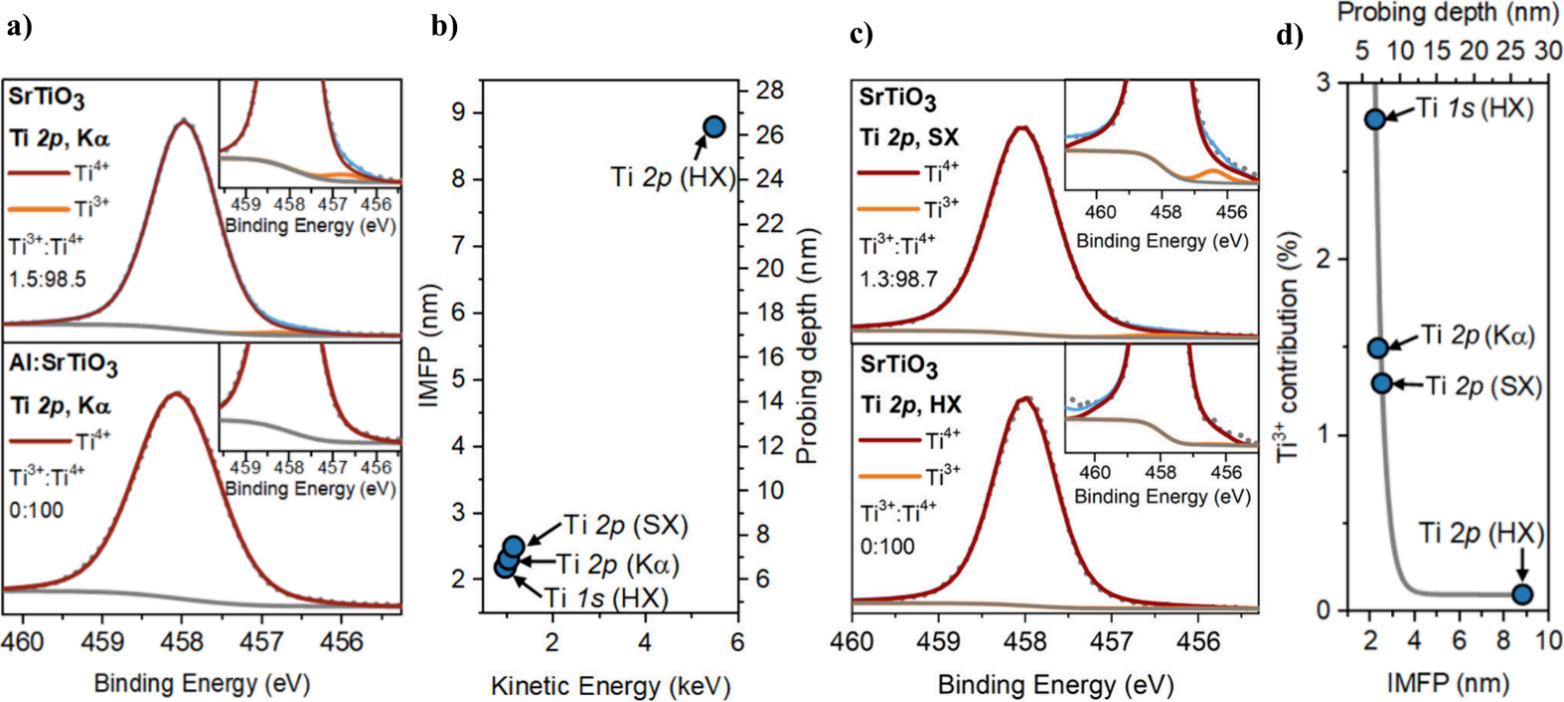
a) Comparison of the core line Ti 2p_3/2_ XPS spectra measured using Al Kα irradiation, including the Ti^3+^ to Ti^4+^ ratio of SrTiO_3_ and Al:SrTiO_3_, with the insets showing the Ti^3+^ contribution to the peak in SrTiO_3_ and the absence of a Ti^3+^ contribution in Al:SrTiO_3_. The dotted gray lines and solid blue lines represent the total measured intensity and the fitted envelope. b) IMFP and the probing depth as a function of kinetic energy, showing the range of penetration depths achieved by the different X-ray measurements. c) Ti 2p_3__/2_ spectra of SrTiO_3_ measured using synchrotron-based XPS employing soft X-rays (SX, 1.6 keV) and HAXPES employing hard X-rays (HX, 5.9 keV) on a binding energy scale calibrated to the Kα Ti 2p_3/2_ peak, with the inset showing the clear Ti^3+^ contribution present using the former. d) Ti^3+^ contribution to the Ti 2p_3/2_ spectrum at different IMFPs and probing depths for SrTiO_3_, noting that the gray line is to illustrate the implied trend.

To elucidate the distribution of Ti^3+^ states below the SrTiO_3_ particle surface, synchrotron-based XPS measurements using soft (*hν* = 1.6 keV, SX) and hard (*hν* = 5.9 keV, HX) X-ray photons were undertaken to provide two additional data points alongside the data generated by the laboratory-based SXPS measurements (*hν* = Kα (∼1.5 keV)). The probing depth of XPS is determined by the inelastic mean free path (IMFP) of the photoelectrons emitted from the sample (probing depth is ∼3 × IMFP, see SI for details), which limits how far into the sample the analysis can effectively capture chemical information. Increasing the X-ray photon energy increases the kinetic energy of emitted photoelectrons, increasing the IMFP and, consequently, the probing depth. As shown in Figure 2b, accessing the Ti 2p core level using the three available photon energies (hν = Kα (∼1.5 keV), 1.6 keV, 5.9 keV) captures information at approximate depths of 6–27 nm (i.e., 2–9 nm IMFP), which is a sufficient probing depth range to achieve a suitable depth dependence study across the particle surface. The additional benefit of the hard X-ray regime is that it unlocks access to the Ti 1s core level. Given the high binding energy of Ti 1s (∼4,963 eV), accessing it with a 5.9 keV X-ray energy makes the information obtained in the Ti 1s core level highly surface sensitive, even more so than conventional laboratory-based SXPS.

When measured with soft X-rays, the Ti 2p_3/2_ peak of SrTiO_3_ has a shoulder toward lower binding energies that corresponds to a Ti^3+^ concentration of 1.3 rel. at.% (as estimated by the peak fits). This contribution is observed to be largely suppressed when measured with hard X-rays (Figure 2c). These Ti^3+^ concentrations, combined with those obtained from the hard X-ray Ti 1s (Figure S7) and laboratory Ti 2p spectra (Figure 2a), indicate a steep decrease in the Ti^3+^ concentration with increasing depths probed (Figure 2d). This suggests that the Ti^3+^ states are concentrated at the surface of SrTiO_3_. While this strong increase in Ti^3+^ concentration at the particle surface may be associated with a greater density of surface oxygen vacancies, it may also be associated with band bending resulting from surface facet energies. Previous studies have highlighted the importance of different surface or facet energies in enabling the high performance of SrTiO_3_ photocatalysts,^30^ as we discuss further below. In any case, these data together indicate that Al^3+^ doping significantly suppresses the density of Ti^3+^ states, particularly at the photocatalyst surface, which is expected to impact the transport, recombination, and reactivity of photogenerated charges.

### Charge Carrier Dynamics of SrTiO_3_ and Al:SrTiO_3_

To elucidate the role of Al^3+^ doping and cocatalyst deposition in enhancing photocatalyst performance, their impact on *in situ* charge carrier dynamics was investigated. We begin by investigating the impact of Al^3+^ doping on quasi-steady state PIAS measurements using 5–10 s pulsed LED irradiation and measuring in diffuse reflectance mode due to the scattering nature of the materials (see details in SI). When the LED excitation is turned on in a PIAS measurement, long-lived photogenerated charges can accumulate in the photocatalyst. This leads to an increase in the signal until a plateau is observed, corresponding to a steady-state charge density being reached. Upon Al^3+^ doping, the PIAS amplitude of SrTiO_3_ increases 5-fold at 500 nm (Figure 3a), with an average 3-fold increase observed across the spectrum (Figure S8). This represents a significant increase in the steady-state charge density achieved, without the addition of strong anodic bias or scavengers, and is indicative of the intrinsic properties of Al:SrTiO_3_ resulting in greater charge lifetimes and accumulation. Meanwhile, the spectral assignments from scavenger studies (whereby the photocatalyst sheets are submerged in 2-propanol as a “hole scavenger” to identify which charge signals correspond to reactive holes) are unchanged following Al^3+^ doping. In both SrTiO_3_ and Al:SrTiO_3_, signals observed <650 nm and in the early NIR are primarily assigned to holes and electrons, respectively (Figures S9 and S10), in good agreement with our recent spectroscopic study of SrTiO_3_ under applied bias^21^ and spectral assignments from previous spectroscopic studies.^10,21,23,24^ The enhancement in PIAS signal amplitude with Al^3+^ doping is most pronounced for wavelengths <600 nm, assigned to valence band holes. As such, these PIAS data clearly indicate that Al^3+^ doping significantly enhances the accumulation of long-lived holes under quasi-steady state irradiation, consistent with its suppression of the density of surface Ti^3+^ states, as evidenced by the XPS data discussed above.

**Figure 3:**
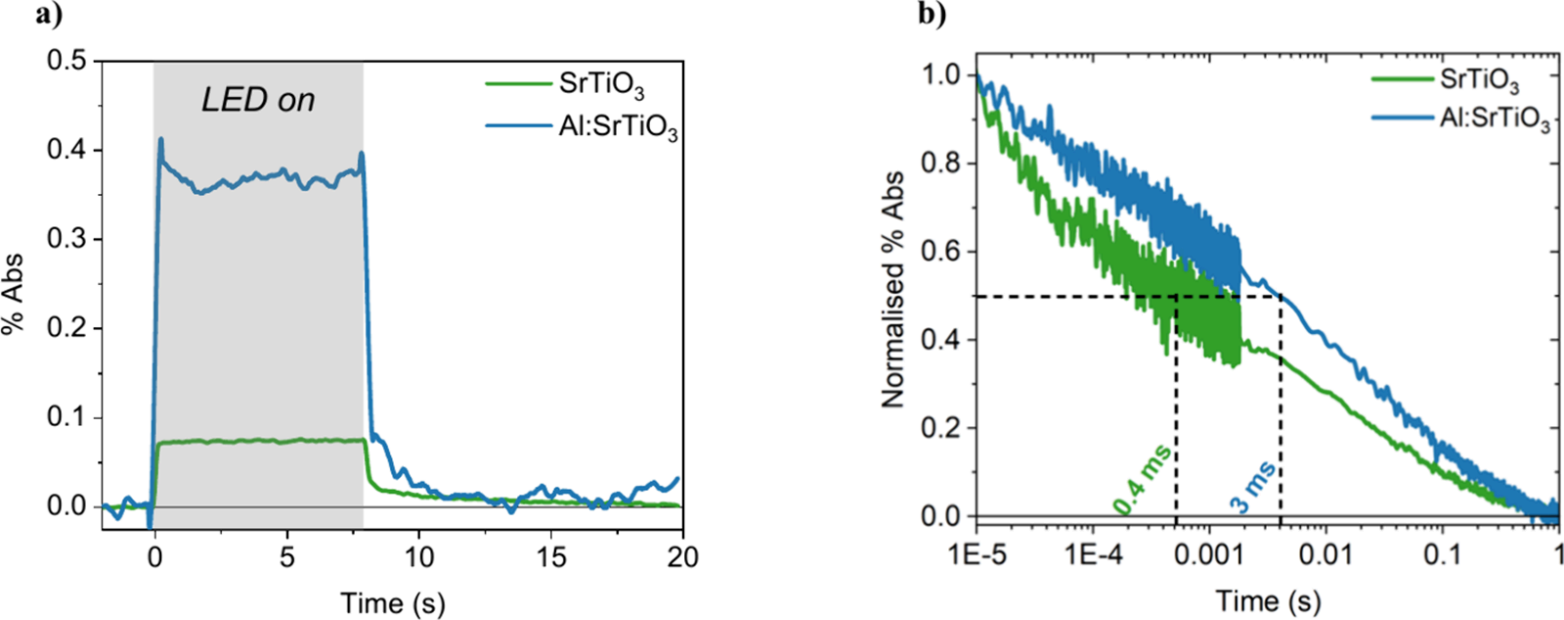
a) PIAS kinetics of SrTiO_3_ and Al:SrTiO_3_ in H_2_O measured using a 365 nm LED at an intensity equivalent to 1 sun illumination (∼2.7 mW cm^–2^) . b) DRTS kinetics of SrTiO_3_ and Al:SrTiO_3_ in H_2_O probed at 500 nm, measured using 355 nm laser excitation at an intensity of 400 μJ cm^–2^ and a 0.8 Hz repetition rate.

The charge carrier dynamics of SrTiO_3_ and Al:SrTiO_3_ were compared on earlier (μs–s) time scales by DRTS. These studies employed short (∼ns) pulsed laser excitations, which result in higher initial carrier densities and faster recombination kinetics than the *in situ* PIAS measurements discussed above. In Al:SrTiO_3_, slower decays and a significant increase in signal amplitude are observed across the spectrum compared to SrTiO_3_ (Figure 3b, Figure S11). For example, the hole lifetimes (*t*_50%_) probed at 500 nm are increased from 0.4 ms in SrTiO_3_ to 3 ms in Al:SrTiO_3_. This increase in hole lifetime with Al^3+^ doping is in agreement with analogous studies by Murphy et al.^10^ The slower DRTS decays in Al:SrTiO_3_, in addition to the increased signal amplitudes measured in DRTS and PIAS, highlight the ability of Al:SrTiO_3_ to support larger charge carrier densities and lifetimes. This can be attributed to the combined effects of suppressing an otherwise high density of Ti^3+^ defect states and introducing a more faceted morphology, as evidenced by the SEM and XPS data discussed above. It is apparent that together these effects can efficiently stabilize charge separation and suppress recombination.^6,31–33^

In addition to increasing charge lifetimes, further effects of Al^3+^ doping are observed from PIAS transients as a function of the light irradiation intensity. In SrTiO_3_, the PIAS decays are independent of the intensity (Figure 4a). This is typical of a monomolecular decay process where one charge is in excess, such as pseudo-first-order recombination with the majority electron carriers (i.e., Ti^3+^ species) being in excess in this case.^34^ We have recently reported analogous intensity (and bias) independent recombination kinetics in sputter-deposited SrTiO_3_ photoelectrodes.^21^ In contrast, the Al:SrTiO_3_ decays accelerate with increasing intensity (Figure 4b), which is a common observation for the decay of reactive charges in metal oxides,^20–22^ and can be assigned to increased bimolecular recombination or reaction rates with increasing charge density. This observation is supported by the intensity dependence of the DRTS decays, whereby the decays of SrTiO_3_ are intensity independent while those of Al:SrTiO_3_ accelerate slightly with increasing intensity (Figure S12). The change from intensity independent monomolecular decays in SrTiO_3_ to intensity dependent decays in Al:SrTiO_3_ is consistent with the suppression of Ti^3+^ donor states detailed above.

**Figure 4:**
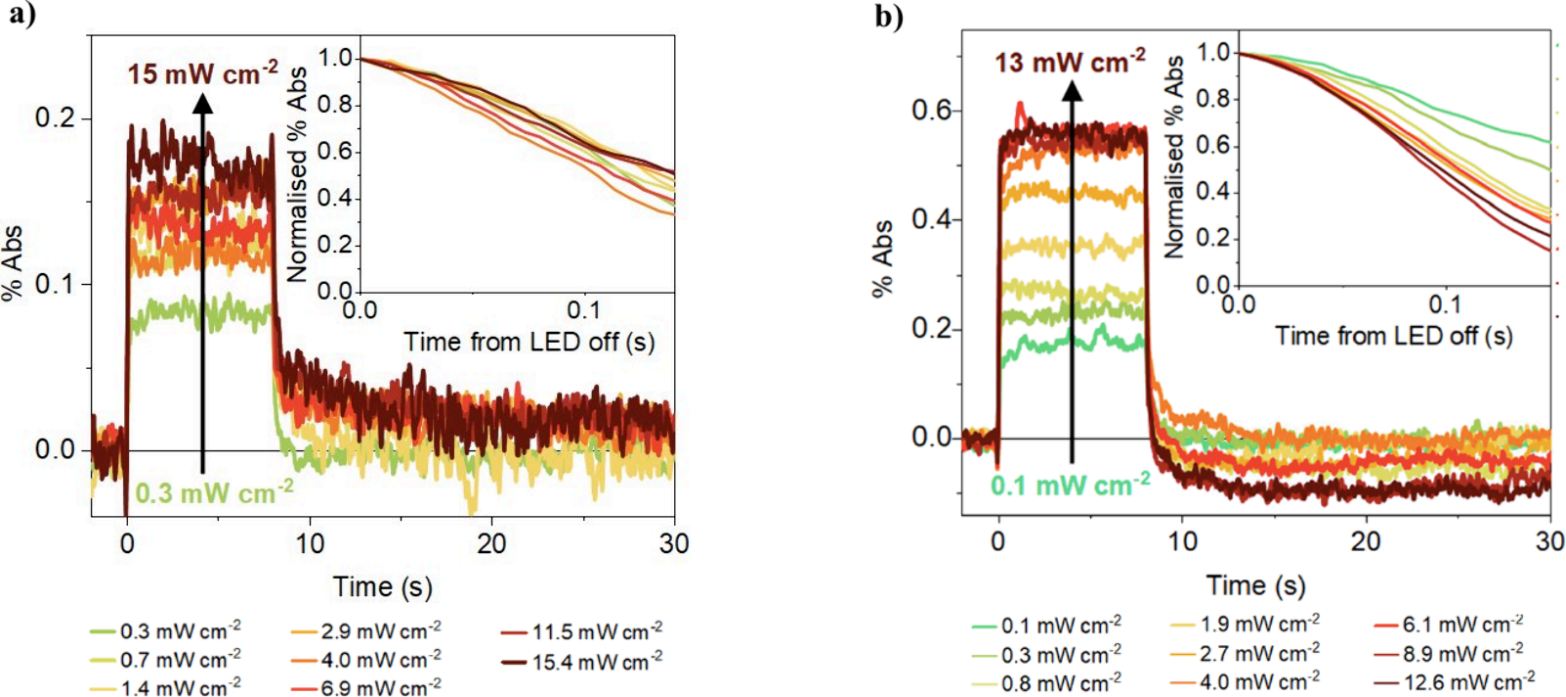
Intensity dependence of the PIAS traces using a 365 nm LED and probed at 500 nm in H_2_O, with insets showing the normalized decay kinetics of the PIAS signal following light-off, for a) SrTiO_3_ and b) Al:SrTiO_3_.

### Charge Carrier Dynamics of Al:SrTiO_3_/RhCrO_*x*_(IMP) and Al:SrTiO_3_/Rh­(PD)/Cr_2_O_3_ Water Splitting Photocatalysts

Now we turn to the impact of Rh–Cr-based cocatalyst deposition on the steady-state charge carrier dynamics, focusing initially on the impregnation deposition. In contrast to the PIAS decays of SrTiO_3_ and Al:SrTiO_3_, which are almost entirely dominated by a relatively fast decay phase (<1 s decay times), the Al:SrTiO_3_/RhCrO_*x*_(IMP) exhibits a larger amplitude and longer lived PIAS signal (Figure 5a). This is indicative of Rh–Cr-based cocatalyst deposition increasing the yield/lifetime of photogenerated holes, attributed to the efficient extraction of photogenerated electrons by RhCrO_*x*_.^35^

**Figure 5:**
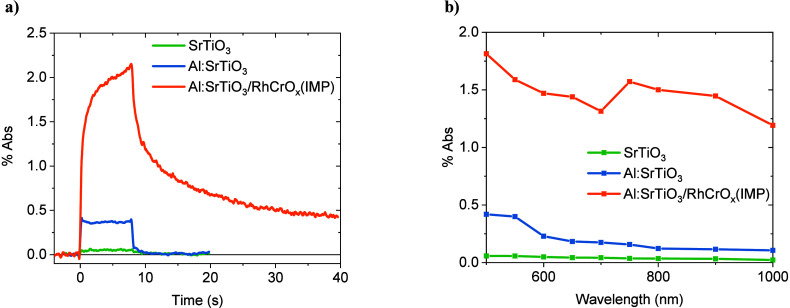
a) PIAS kinetics probed at 500 nm and b) PIAS spectra measured at 8 s of SrTiO_3_, Al:SrTiO_3_, and Al:SrTiO_3_/RhCrO_*x*_(IMP), measured in H_2_O and using a 365 nm LED at an intensity equivalent to 1 sun illumination.

It is apparent that both the rise and decay dynamics of the PIAS signal for Al:SrTiO_3_/RhCrO_*x*_(IMP) in Figure 5a appear biphasic, with “fast” (<1 s) and “slow” (10s of seconds) phases; further discussion and analyses of these distinct phases are given below. The addition of a hole scavenger suppresses the signal amplitude for Al:SrTiO_3_/RhCrO_*x*_(IMP) to an equivalent degree across the spectrum (Figure S13), indicative of holes being probed across all wavelengths and electrons no longer dominating at longer wavelengths. This is attributed to the faster time scales reported for electron transfer to the Rh–Cr-based cocatalyst (subμs),^10,35^ as well the potential for subsequent proton reduction (μs–ms).^36,37^ Interestingly, in the presence of a hole scavenger, the fast decay phase is accelerated and becomes more dominant, while the slow phase kinetics are unchanged (Figure S14). Thus, the fast and slow phases are assigned to reactive and unreactive hole species, respectively (see the following section for further justification). This assignment is consistent with a recent study by Chen et al., who observed similar fast decay phases of hole species in SrTiO_3_ photoanodes under water oxidation conditions^30^ and also the assignment of analogous fast/slow PIAS decays on BiVO_4_ photocatalysts.^38^ With deep trap states previously observed in SrTiO_3_ photoanodes and hole trapping linked to its slow PIAS decay phase,^21^ we assign the slow decay phase to the accumulation of deeply trapped holes, consistent with their relative unreactivity.

In contrast to Al:SrTiO_3_/RhCrO_*x*_(IMP), the decay kinetics of Al:SrTiO_3_/Rh­(PD)/Cr_2_O_3_ are dominated by the fast decay phase (Figure 6a, with non-normalized kinetics shown in Figure S15), with the slow phase being of lower relative amplitude. This is indicative of the accumulation of a greater proportion of more reactive holes on Al:SrTiO_3_/Rh­(PD)/Cr_2_O_3_ and the suppression of deep hole trap states, which is consistent with its higher AQY for overall water splitting. In addition to accelerated decay kinetics, the signal amplitudes in Al:SrTiO_3_/Rh­(PD)/Cr_2_O_3_ are suppressed compared to those in Al:SrTiO_3_/RhCrO_*x*_(IMP) (Figure S16), attributed to the faster hole reaction kinetics reducing the steady state density of accumulated holes.

**Figure 6:**
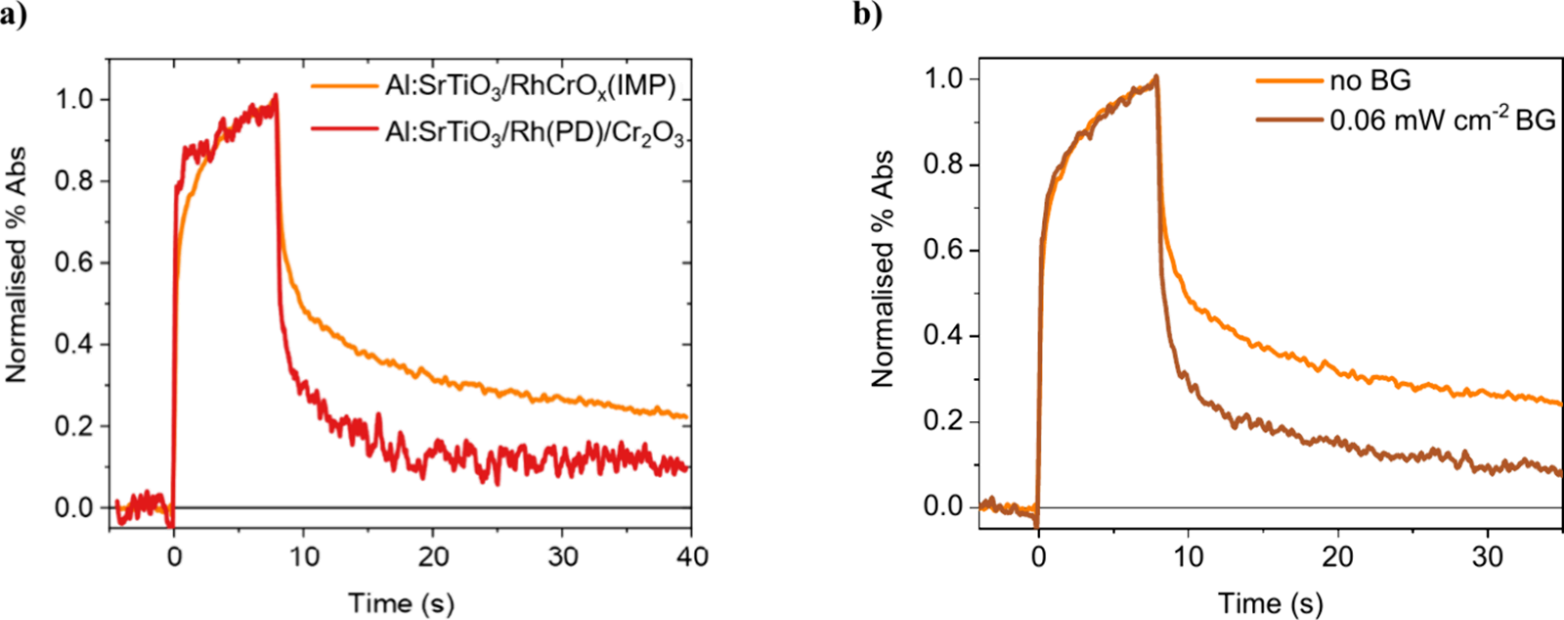
Comparison of the PIAS kinetics measured using a 365 nm LED at an intensity equivalent to 60% sun illumination and probed at 500 nm in H_2_O, of a) Al:SrTiO_3_/RhCrO_*x*_(IMP) and Al:SrTiO_3_/Rh­(PD)/Cr_2_O_3_ and of b) Al:SrTiO_3_/RhCrO_*x*_(IMP) with and without continuous background illumination from a second 365 nm LED. Note that these measurements were undertaken at 60% sun illumination, as intense bubble formation from Al:SrTiO_3_/Rh­(PD)/Cr_2_O_3_ interfered too significantly with spectroscopic measurements under 1 sun illumination.

Further insight into the hole dynamics on Al:SrTiO_3_/RhCrO_*x*_(IMP) and Al:SrTiO_3_/Rh­(PD)/Cr_2_O_3_ was obtained by employing background illumination and a range of excitation intensities. In both Al:SrTiO_3_/RhCrO_*x*_(IMP) and Al:SrTiO_3_/Rh­(PD)/Cr_2_O_3_, the fast PIAS decay phase accelerates with increasing intensity (Figure S15), consistent with previous studies of water oxidation kinetics on photoanodes of SrTiO_3_ ^30^ and other metal oxides^39–41^ and therefore supporting the assignment of this decay phase to water oxidation. Continuous background illumination of Al:SrTiO_3_/RhCrO_*x*_(IMP) from an additional 365 nm LED accelerates the PIAS decay kinetics and suppresses the proportion of slow phase decay assigned to deeply trapped holes (Figure 6b). The acceleration of decay dynamics is consistent with overall higher charge accumulation. In particular, the suppression of the slow phase in Al:SrTiO_3_/RhCrO_*x*_(IMP) under background illumination is indicative of this background illumination filling the deep hole trap states, effectively passivating them and suppressing further hole trapping. This behavior is analogous to previous reports of preillumination suppressing deep electron trapping in ionic carbon nitrides and BiVO_4_ ^38,42^ and is also analogous to illumination passivating trap states in organolead halide perovskite solar cells.^43^ The impact of background illumination on Al:SrTiO_3_/Rh­(PD)/Cr_2_O_3_ is significantly suppressed compared to that on Al:SrTiO_3_/RhCrO_*x*_(IMP) (Figure S17), consistent with a lower density of deep trap states in Al:SrTiO_3_/Rh­(PD)/Cr_2_O_3_.

## Discussion

### Origins and Role of Long-Lived Hole Accumulation

Water oxidation on metal oxide photocatalysts and photoelectrodes is a kinetically slow process, typically proceeding on the 100 ms–10 s time scale. It therefore requires the accumulation of commensurately long-lived oxidizing species (valence band holes for semiconducting oxides) to drive this reaction. In the study herein, we have employed *in situ*/*operando* photoinduced absorption spectroscopy to measure the accumulation and kinetics of such holes on SrTiO_3_ photocatalyst particles. We observe that flux-mediated Al^3+^ doping results in a 5-fold increase in the accumulation of SrTiO_3_ holes under quasi-steady state one sun irradiation, as well as a 7-fold increase in their lifetime, correlated with the suppression of surface Ti^3+^ species observed by XPS. The deposition of a Rh–Cr-based proton reduction cocatalyst is observed to further increase long-lived hole accumulation, as well as increase the lifetime of these accumulated holes. This is assigned to efficient electron extraction by the Rh–Cr-based cocatalysts, spatially separating charge to reduce electron/hole recombination and enhance the steady state charge lifetimes and densities, as we observe herein, as well as suppressing the unwanted oxygen reduction reaction. Two distinct hole populations are observed: a fast (ca. 1 s) decaying population assigned to holes driving water oxidation, and a slow (>10 s) decaying population assigned to deeply trapped and, therefore, unreactive holes. Both impregnation and photodeposition routes to depositing Rh–Cr-based cocatalysts were investigated, with photodeposition suppressing the slow hole decay phase assigned to deep hole trapping, indicative of a higher yield of reactive holes and consistent with the higher AQY observed for overall water splitting.

While Al^3+^ doping and the Rh–Cr-based cocatalysts are integral to achieving the state-of-the-art water splitting performance of the Al:SrTiO_3_-based systems,^15,18^ the intrinsic properties of SrTiO_3_ are also expected to play a key role in enabling charges to accumulate and persist without a concurrent increase in recombination. For example, the notably slow bimolecular recombination of SrTiO_3_ ^10,19,20^ minimizes the recombination of photogenerated charges and maximizes the yields available for the slow interfacial water splitting reactions. In addition, the deep valence band of SrTiO_3_ relative to the potential of water oxidation offers a large overpotential to drive this kinetically slow multiredox reaction.

### Synergistic Roles of Flux-Mediated Al^3+^ Doping and Rh–Cr-Based Cocatalyst Addition in Enabling State-of-the-Art Photocatalytic Performance

Our data herein demonstrate the key role of flux-mediated Al^3+^ doping in suppressing charge recombination and increasing the yield of long-lived charges in SrTiO_3_. The origin of this enhancement is potentially 3-fold. First, Al^3+^ doping suppresses the high density of Ti^3+^ states in SrTiO_3_ that can otherwise serve as recombination centers,^10–12,29^ as evidenced by our XPS data. Second, the Al^3+^ doping procedure introduces a faceted morphology, which has been suggested to generate an internal electric field between facets to spatially separate electrons and holes, and enables the selective photodeposition of Rh onto reduction facets in Al:SrTiO_3_/Rh­(PD)/Cr_2_O_3_.^9^ Finally, the suppressed donor density resulting from Al^3+^ doping decreases screening of the facet induced electric field, enabling this field to penetrate further into the bulk of each particle to drive the spatial separation of charges.^9,44^ The above effects of Al^3+^ doping are summarized in Figure 7a and 7b. In SrTiO_3_, the absence of an internal electric field and the high density of Ti^3+^ dopant states result in relatively rapid charge recombination. Charge recombination via these Ti^3+^ dopant states is supported by our observation of intensity independent (i.e.: pseudo-first order) millisecond recombination kinetics under pulsed laser excitation (Figure S12). The Ti^3+^ states are assigned to filled electron trap states, most likely associated with oxygen vacancies, as discussed in our previous work.^21^ In Al:SrTiO_3_, the suppression of Ti^3+^ states suppresses this trap/dopant-mediated recombination pathway (Figure 7b). In addition, the increased particle faceting introduces an internal electric field to spatially separate electrons and holes, further suppressing recombination losses and enabling the enhanced accumulation of long-lived charges. For simplicity, we assume a uniform field, although we note that the formation of space charge layers may result in a field-free region in the particle interior. In any case, the suppression of Ti^3+^ and the facet-induced field results in an increase in hole lifetime and thus enhanced hole accumulation.

**Figure 7:**
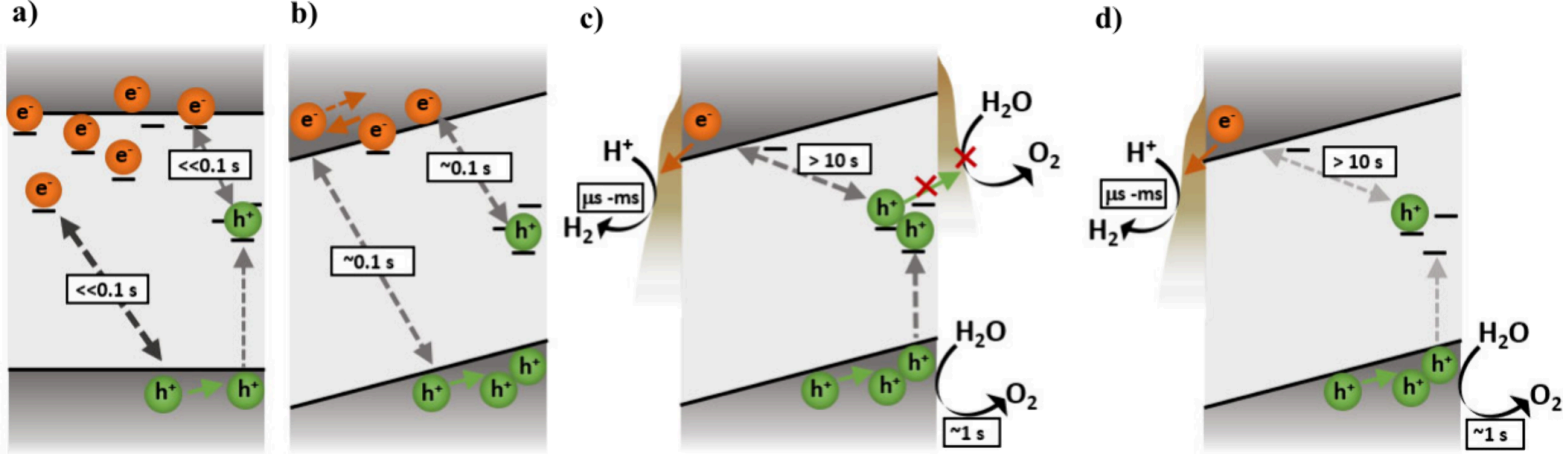
Schematic illustration of the proposed recombination, trapping, and function of a) SrTiO_3_, b) Al:SrTiO_3_, c) Al:SrTiO_3_/RhCrO_*x*_(IMP), and d) Al:SrTiO_3_/Rh­(PD)/Cr_2_O_3_, under steady-state irradiation for water splitting. The sloped conduction band and valence band edges following Al^3+^ doping in b) onward represent the internal electric field introduced, driving charge separation. In d) (Al:SrTiO_3_/Rh­(PD)/Cr_2_O_3_), RhCrO_*x*_ is selectively deposited on the reduction facets, whereby electrons are extracted to RhCrO_*x*_ for proton reduction. However, in c) (Al:SrTiO_3_/RhCrO_*x*_(IMP)), RhCrO_*x*_ is deposited on both the reduction and oxidation facets, proposed to inhibit proton reduction when on the oxidation facet and result in a higher population of deeply trapped holes. The >10 s decay time in Al:SrTiO_3_/RhCrO_*x*_(IMP) and Al:SrTiO_3_/Rh­(PD)/Cr_2_O_3_ represents the slow decay phase between deeply trapped holes and conduction band electrons, while the fast decay phase is represented by the ∼1 s water oxidation reaction.

Despite the suppression of charge recombination observed for Al:SrTiO_3_, our PIAS data indicate that, in the absence of a hydrogen evolution cocatalyst, these charges still decay with a half-time of ∼100 ms. Comparison with the time scale for water oxidation reported previously for both SrTiO_3_ and other metal oxides^21,45^ suggests this 100 ms lifetime is too short to drive efficient water oxidation and is therefore most likely to be dominated by recombination losses. This is consistent with the low water splitting activity observed for these photocatalysts in the absence of added cocatalysts. As such, it is striking that Rh–Cr-based cocatalyst deposition results in a further increase in both the yield and lifetime of photogenerated holes. This is attributed to fast electron extraction to the Rh–Cr-based cocatalyst (sub-μs, prior to the PIAS measurement time scale),^35^ further enhancing the spatial charge separation, as illustrated in Figure 7c and 7d. The resultant accumulated holes are subsequently observed to exhibit a dominant ∼1 s decay phase, long enough to drive efficient water oxidation and consistent with the high AQYs observed in the presence of this cocatalyst.

We note that the addition of CoOOH has been reported to further enhance the performance of Al:SrTiO_3_ loaded with Rh–Cr-based cocatalysts.^15^ PIAS studies of Al:SrTiO_3_/RhCrO_*x*_(IMP) in the presence and absence of CoOOH did not resolve a clear difference in the PIAS spectrum or decay kinetics (Figure S18). This suggests that CoOOH deposition has only a minor impact on the charge carrier dynamics compared to the Rh–Cr-based cocatalysts, consistent with observations that CoOOH deposition primarily enhances photocatalyst stability rather than quantum efficiency. This may be related to the relatively slow kinetics of water oxidation on CoOOH, with an order of magnitude similar to those observed herein for SrTiO_3_,^46^ although detailed analysis of these points is beyond the scope of this study.

### Notable Features of Overall Water Splitting Photocatalytic Systems

The ability of Al:SrTiO_3_ loaded with Rh–Cr-based cocatalysts to generate high yields of long-lived charges is central to their high photocatalytic performance. We note that the scattering nature of these samples prevents us from quantifying the yield of charges, although we do note that the signal sizes observed (up to 2.5% Abs) is indicative of accumulated hole densities comparable to high performance photoanodes under strong anodic bias.^45^ This ability of Al:SrTiO_3_ loaded with Rh–Cr-based cocatalysts to achieve efficient long-lived charge accumulation is attributed to the combined effects of the Rh–Cr-based proton reduction cocatalyst, Al^3+^ doping, and the intrinsic SrTiO_3_ properties. We have observed a correlation between long-lived charge accumulation and efficiency in other photocatalytic systems, although the underlying mechanisms are unique in each case.^35,47^ For example, in GaN:ZnO modified with RhCrO_*x*_(IMP), the energy offset between the GaN and ZnO phases is thought to be an important factor, with electron extraction to RhCrO_*x*_(IMP) enhancing the separation.^35^ Meanwhile, in organic heterojunctions for hydrogen evolution, long-lived charges were correlated to higher photocatalytic activities and attributed, at least in part, to their phase-segregated morphology.^47^ Regardless of the mechanisms underpinning these species, the fact that they are frequently observed in efficient photocatalytic systems, but only observed in photoanodes under applied bias, suggests that the ability to accommodate long-lived species in the absence of applied bias is an important design feature for efficient photocatalysts.

### Impact of Rh–Cr-Based Cocatalyst Deposition via Facet-Selective Photodeposition Compared to Impregnation

We turn now to a consideration of the difference between Al:SrTiO_3_/RhCrO_*x*_(IMP) and Al:SrTiO_3_/Rh­(PD)/Cr_2_O_3_. For both photocatalysts, in addition to the ∼1 s decay phase assigned to reactive holes driving water oxidation, a slower (∼10 s) PIAS decay phase, assigned to a low yield of deeply trapped and unreactive hole species, was also observed (Figure 7c and 7d). It is evident from their long lifetime that these deeply trapped holes do not function as rapid recombination centers, most likely due to the presence of facet band bending and the efficient extraction of photogenerated electrons by Rh–Cr-based cocatalysts. This deeply trapped hole signal is suppressed for Al:SrTiO_3_/Rh­(PD)/Cr_2_O_3_ compared to Al:SrTiO_3_/RhCrO_*x*_(IMP), which is likely to be a factor in the lower AQY achieved with impregnation rather than photodeposition.^15^ The precise chemical identity of these deeply trapped hole states is uncertain; however, they may result from defect states, such as oxygen vacancies in anisotropic SrTiO_3_.^29^ These origins are supported by continuous background illumination suppressing the slow decay phase, indicating that these trap states can be passivated (i.e., oxidized) by illumination, increasing the yield of reactive holes, as reported previously in other photocatalysts including BiVO_4_ ^38,41,42^ and organolead halide perovskites.^43^ This passivation is also consistent with our previous report for Al:SrTiO_3_/RhCrO_*x*_(IMP) of a slow initial rate of oxygen evolution which increased under illumination until a steady state was achieved after several seconds.^48^ Our PIAS data herein suggest that this lag in oxygen evolution may result from an initial illumination-driven filling of deep hole trap states, which results in their passivation and subsequent enhanced photocatalytic performance.

An alternative explanation for the origins of the deep hole trap states is that the electron accumulation on the reduction facets structurally alters the surface structure, such that it increases the presence and population of deeply trapped hole states. This effect is exacerbated in Al:SrTiO_3_/RhCrO_*x*_(IMP) compared to Al:SrTiO_3_/Rh­(PD)/Cr_2_O_3_, as demonstrated by the greater prevalence of the long-lived hole signal assigned to deeply trapped hole states. This is supported by the higher contribution of metallic Rh in Al:SrTiO_3_/Rh­(PD)/Cr_2_O_3_ (Figure S6) having a greater ability to dissipate electrons via proton reduction than Rh oxide, which is more dominant in Al:SrTiO_3_/RhCrO_*x*_(IMP).

While the chemical identity of these deep hole trap states is uncertain, it is apparent that photodeposition of Cr_2_O_3_/Rh rather than impregnation results in a suppression of these states. In Al:SrTiO_3_/RhCrO_*x*_(IMP), RhCrO_*x*_(IMP) is randomly distributed across both the reduction and oxidation facets. RhCrO_*x*_(IMP) deposition on the oxidation facets is likely to inhibit efficient water oxidation (Figure 7c). In particular, holes could become trapped in (i.e., recombine with) RhCrO_*x*_(IMP), where they can oxidize Cr^3+^ to Cr^6+^ and deactivate the cocatalyst.^18^ In either case, it is likely that these deep trap states are associated, at least in part, with undesirable Rh–Cr-based cocatalyst deposition on oxidation facets, consistent with the high photocatalytic performance observed following facet-selective photodeposition.

## Conclusion

In this work, we have focused on the use of *in situ*/*operando* time-resolved spectroscopy to monitor the accumulation and dynamics of photogenerated holes critical to the remarkably efficient function of Al:SrTiO_3_ photocatalysts loaded with Rh–Cr-based cocatalysts. Flux-mediated Al^3+^ doping is observed to increase the accumulation of long-lived (∼100 ms) charges. This is attributed to this doping exploiting the chemical flexibility of the SrTiO_3_ perovskite structure to suppress Ti^3+^ recombination centers and introduce a faceted morphology which generates an internal electric field to drive the spatial separation of charges. Subsequent Rh–Cr-based cocatalyst deposition further increases the yield and lifetime of photogenerated holes, with a dominant, light intensity dependent, ∼1 s decay phase assigned to water oxidation. This increase in hole yield and lifetime is attributed to efficient electron extraction to Rh–Cr-based cocatalysts, increasing the spatial separation of charges. An additional, low amplitude ∼10 s decay phase is also observed and assigned to deeply trapped and unreactive holes. The yield of these deeply trapped holes is suppressed by background illumination—indicative of trap filling passivating these states. The yield of these deeply trapped states is further suppressed for photodeposited rather than impregnated Rh–Cr-based cocatalysts. Thus, it is suggested that this suppression of deep hole trapping may be an additional factor behind the near-*unity* AQY achieved for Al:SrTiO_3_/Rh­(PD)/Cr_2_O_3_. As such, the study herein highlights the impact of the key design features of these state-of-the-art photocatalysts—including the use of chemical dopants (i.e., Al^3+^) to suppress the electrical doping (i.e., Ti^3+^) density, faceting to drive the spatial separation of charges, and cocatalyst deposition to stabilize this charge separation—on the generation of the long-lived holes required to drive the key, but kinetically slow, water oxidation reaction. These design features are concepts that can be applied in the development of narrow band gap photocatalysts that can simultaneously achieve enhanced visible light absorption and a high AQY.

## Supplementary Material



## Notes

Experimental details of the materials synthesis, in addition to SEM, XRD, XPS, HAXPES, PIAS, and DRTS measurements. Supplementary figures include: XRD patterns, UV–vis absorption spectra, Tauc plots, additional XPS spectra not included in the manuscript, comparison of PIAS spectra of SrTiO_3_ and Al:SrTiO_3_ in H_2_O and in 2-propanol, comparison of DRTS spectra of SrTiO_3_ and Al:SrTiO_3_ in H_2_O, DRTS kinetics of SrTiO_3_ and Al:SrTiO_3_ in H_2_O, comparison of PIAS spectra of Al:SrTiO_3_/RhCrO_*x*_(IMP) and Al:SrTiO_3_/Rh­(PD)/Cr_2_O_3_ in H_2_O and in 2-propanol, additional intensity dependence kinetics of PIAS kinetics, comparison of PIAS spectra and kinetics of Al:SrTiO_3_/RhCrO_*x*_(IMP) and Al:SrTiO_3_/Rh­(PD)/Cr_2_O_3_ with and without background illumination (PDF)
