# Supplementary material for: Long-Lived Hole Accumulation in Al:SrTiO3/Rh–Cr Photocatalyst Systems under Continuous Irradiation and Its Correlation with Overall Water Splitting Efficiency
Source: J Am Chem Soc. 2025 Sep 11;147(38):34438–48. doi: 10.1021/jacs.5c07521 (PMC12464978; doi:10.1021/jacs.5c07521)
Supplement: Supplementary file 1 [file ja5c07521_si_001.pdf]

Long-lived hole accumulation in Al:$SrTiO_3$/Rh-Cr photocatalyst systems under continuous irradiation and its correlation with overall water splitting efficiency

Anna Wilson,[a] Benjamin Moss,[a,b] Aysha A. Riaz,[c] Curran Kalha,[c] Pardeep K. Thakur,[d] Tien-Lin Lee,[d] Anna Regoutz,[c,e] Tsuyoshi Takata,[f] Takashi Hisatomi,[g] Kazunari Domen,[g,h]* James R Durrant[a,i]*

[a] Department of Chemistry, Centre for Processable Electronics, Imperial College London, London, UK, SW7 2AZ

[b] Resnik Centre for Sustainability, California institute of Technology, Los Angeles, USA, 91125

[c] Department of Chemistry, University College London, 20 Gordon Street, London, UK, WC1E 7HU

[d] Diamond Light Source LTD., Diamond House, Harwell Science and Innovation Campus, Didcot, UK, OX11 0DE

[e] Department of Chemistry, University of Oxford University, Inorganic Chemistry Laboratory, South Parks Road, Oxford, UK, OX1 3QR

[f] Research Initiative for Supra-Materials, Shinshu University, 4-17-1 Wakasato, Nagano-shi, Nagano 380-0928, Japan

[g] Institute for Aqua Regeneration, Research Initiative for Supra-Materials, Shinshu University, 4-17-1 Wakasato, Nagano-shi, Nagano 380-0928, Japan

[h] Office of University Professors, The University of Tokyo, 2-11-16 Yayoi, Bunkyo-ku, Tokyo 113-8656, Japan

[i] Department of Materials Science and Engineering, Swansea University, Swansea, UK, SA2 8PP

Table of contents

Experimental 3
Materials synthesis 3
Materials characterization 3
X-ray photoelectron spectroscopy 3
Time-resolved spectrometry 4
Results 5
References 13

Experimental

Materials synthesis

The $SrTiO_3$-based photocatalyst sheets studied in this work were provided by Domen and co-corkers, where they were fabricated by drop-casting their respective powders with a SiO_2 binder onto quartz substrates. The $SrTiO_3$ powder was doped with Al^{3+} by a flux mediated doping process described in their previous work,[1] using $SrTiO_3$, $SrCl_2$ and Al_2O_3 in a 1:10:0.02 ratio. For the deposition of Rh-Cr-based co-catalysts, either an impregnation or photodeposition method was followed, as explained in detail elsewhere.[2–5]

Materials characterisation

Scanning electron microscopy (SEM), coupled with energy dispersive X-ray analysis (EDX), using a Leo Gemini 1525 Field Emission Gun scanning electron microscope (FEG SEM) was used to reveal the morphology of the photocatalysts following sputtering with a 15 mm Cr coating. X-ray diffraction (XRD) patterns were obtained using a Bruker D2 phaser with parallel beam optics equipped with a PSD LinxEye silicon strip detector, using Cu Kα radiation (40 kV and 40 mA). Ground state reflectance was measured using a UV-Vis spectrometer (Shimadzu UV-2600) fitted with an integrating sphere (Shimadzu ISR-2600Plus). Reflectance data was converted into dimensionless units proportional to absorbance using the Kubelka-Munk function ($F(R)=\frac{(1-R)^2}{2R}$) and the band gaps were derived from Tauc plots from the optical data.

X-ray photoelectron spectroscopy

The chemical composition was investigated by X-ray photoelectron spectroscopy (XPS) methods. Laboratory XPS measurements were undertaken on particulate powder samples on carbon tape, using a Thermo K-Alpha spectrometer with a monochromated Al Kα radiation source. Peaks were calibrated according to the adventitious C 1s peak at 284.5 eV and peak fitting and analysis was conducted using Avantage software (Thermo Scientific v.5.9925).

Soft XPS and hard XPS (HAXPES) measurements were conducted at beamline I09 of the Diamond Light Source. Soft XPS measurements were conducted using a photon energy of 1.5946 keV (abbreviated to 1.6 keV in the manuscript), selected using a 400 lines per mm plane grating monochromator, resulting in a final energy resolution of 370 meV. For hard XPS measurements, a photon energy of 5.9267 keV(abbreviated to 5.9 keV in the manuscript) was used, achieved by selecting a post-monochromator Si(004) channel-cut crystal and a double-crystal Si(111) monochromator, yielding a final energy resolution of 280 meV. The total energy resolution was determined by extracting the 16%–84% width of the Fermi edge of a clean polycrystalline gold foil. Samples were mounted on adhesive carbon tape and key core levels in addition to survey scans were measured. Spectral analysis was performed using Avantage software (Thermo Scientific v.5.9925). Spectra were aligned first to the Fermi edge of a polycrystalline gold foil that was measured on the same experimental run as the samples, and then an additional alignment to the Ti $2p_{3/2}$ binding energy position taken from the soft XPS spectrum. A Scienta Omicron EW4000 hemispherical analyser, featuring a ±28° acceptance angle, was employed at the end station of beamline I09. The base pressure in the analysis chamber remained at 3.5×10^{-10} mbar. To optimise the efficiency of spectral collection, the measurements were carried out under grazing incidence and near-normal emission conditions.

The inelastic mean free path (IMFP) of elastic core level electrons were determined using the QUASES-IMFP-TPP2M v3.0 calculator.[6] The calculations were set to relativistic. Manual adjustments were made so that the appropriate direct bandgap energy and bulk density of our samples were taken into account.

Time-resolved spectrometry

Time-resolved spectroscopic measurements were conducted on a home build set-up for measuring the optical signal in diffuse reflectance mode (see Figure S1 for a schematic illustration of the set-up, including the lenses used to concentrate the diffused light). Reagents were continuously bubbled with N_2, unless otherwise stated. A 100 W Bentham IL1 quartz halogen lamp served as the probe light source, followed by long pass filters (Thorlabs) selected for the desired probe wavelength and an IR filter (H_2O, 5 cm path length) to prevent sample heating. For photoinduced absorption spectroscopy (PIAS), the excitation source was a 365 nm LED connected to a power supply (QL564P, Thurlby Thandar Instruments) to control the light intensity and a MOSFET transistor (STF8NM50N, STMicroelectronics). Where LED intensity is referred to as '1 sun', this equates to ~2.7 mW cm^{-2} for the $SrTiO_3$-based photocatalysts studied herein. A liquid light guide (0.5 cm diameter) directed the LED light onto the photocatalyst sheet (drop-casted powder on quartz substrates) samples, in a quartz cuvette. Following the sample was a series of lenses and mirrors for concentrating and directing the diffuse reflected light through a motorised colour wheel (Thorlabs), followed by a monochromator (Oriel Cornerstone 130), set according to the probe wavelength, and finally onto a silicon photodiode detector (Hamamatsu S3071). In diffuse reflectance mode, the %Abs is calculated from the fractional change in reflected light measured by the photodiode ($\Delta R_{(\lambda,t)}$):

$$\%Abs = 1 - R_{(\lambda,t)} = \frac{R_{(\lambda,t_0)} - R_{(\lambda,t)}}{R_{(\lambda,t_0)}} = \Delta R_{(\lambda,t)}$$

Data from 1 ms was recorded by the DAQ card (NI DAQ 3631). The effects of photoluminescence and scattering from the probe light were corrected for by subtracting the data obtained in the absence of the probe light. Labview software was used for data acquisition.

The same set-up was used for diffuse reflectance transient spectroscopy (DRTS) measurements, with the following modifications. Instead of the LED, the third harmonic of a Nd:YAG laser (OPOTEK Inc, Opolette 355 II, 6 ns pulse width, λ_{ex} = 355 nm) served as the excitation source. Instead of data being collected solely by the DAQ, data at early times (μs - 1 ms) was electronically amplified (Costronics) before being recorded by the oscilloscope (Tektronics DPO3012), whilst data at later times (> 1 ms) was recorded by DAQ card. The two data sets were stitched together to produce a continuous transient decay across the full timescale recorded.

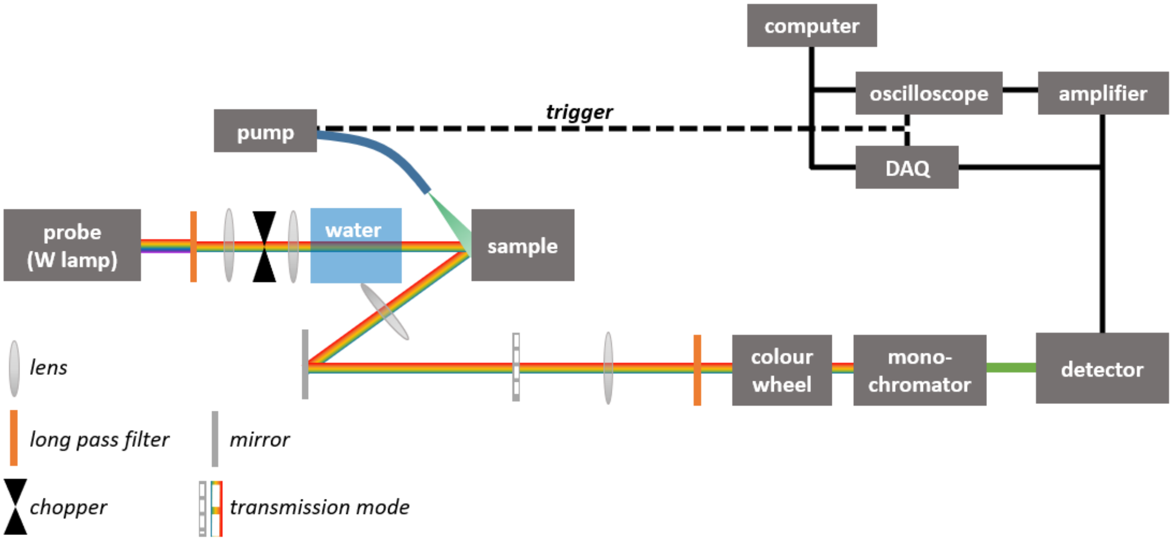

Figure S1: Schematic representation of the transient spectroscopy set-up used to measure samples herein in diffuse reflectance mode.

Results

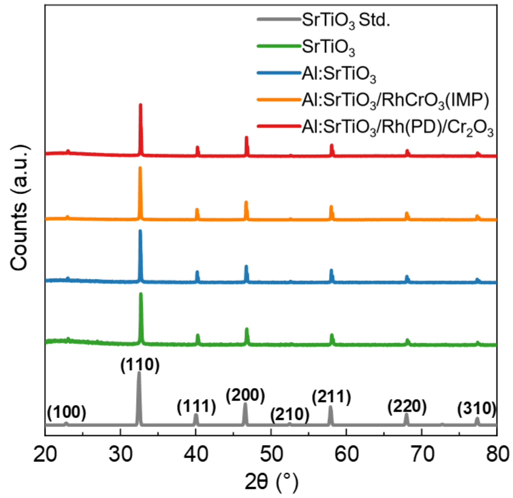

Figure S2: XRD patterns of $SrTiO_3$, Al:$SrTiO_3$, Al:$SrTiO_3$/$RhCrO_x$(IMP) and Al:$SrTiO_3$/Rh(PD)/Cr_2O_3 compared to a $SrTiO_3$ standard (ICSD: 23076).

Table S1: FWHM of the $SrTiO_3$ (110) peak in $SrTiO_3$, Al:$SrTiO_3$, Al:$SrTiO_3$/$RhCrO_x$(IMP) and Al:$SrTiO_3$/Rh(PD)/Cr_2O_3.

	**$SrTiO_3$**	**Al:$SrTiO_3$**	**Al:$SrTiO_3$/$RhCrO_x$(IMP)**	**Al:$SrTiO_3$/Rh(PD)/Cr_2O_3**
**(110) XRD peak FWHM**	0.17	0.13	0.13	0.13

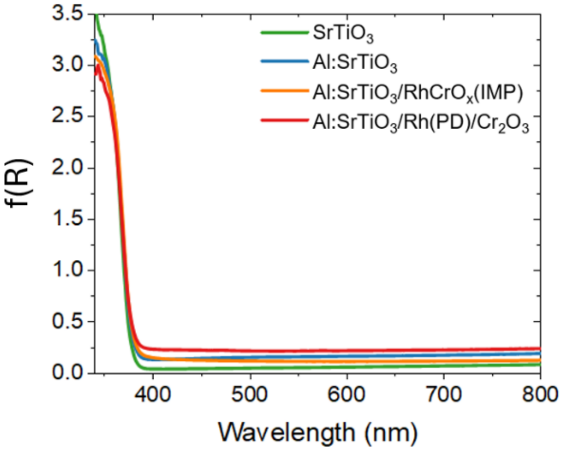

Figure S3: UV-Vis absorption spectra measured using an integrating sphere, of $SrTiO_3$, Al:$SrTiO_3$, Al:$SrTiO_3$/$RhCrO_x$(IMP) and Al:$SrTiO_3$/Rh(PD)/Cr_2O_3, plotted as the Kubelka-Munk function against wavelength.

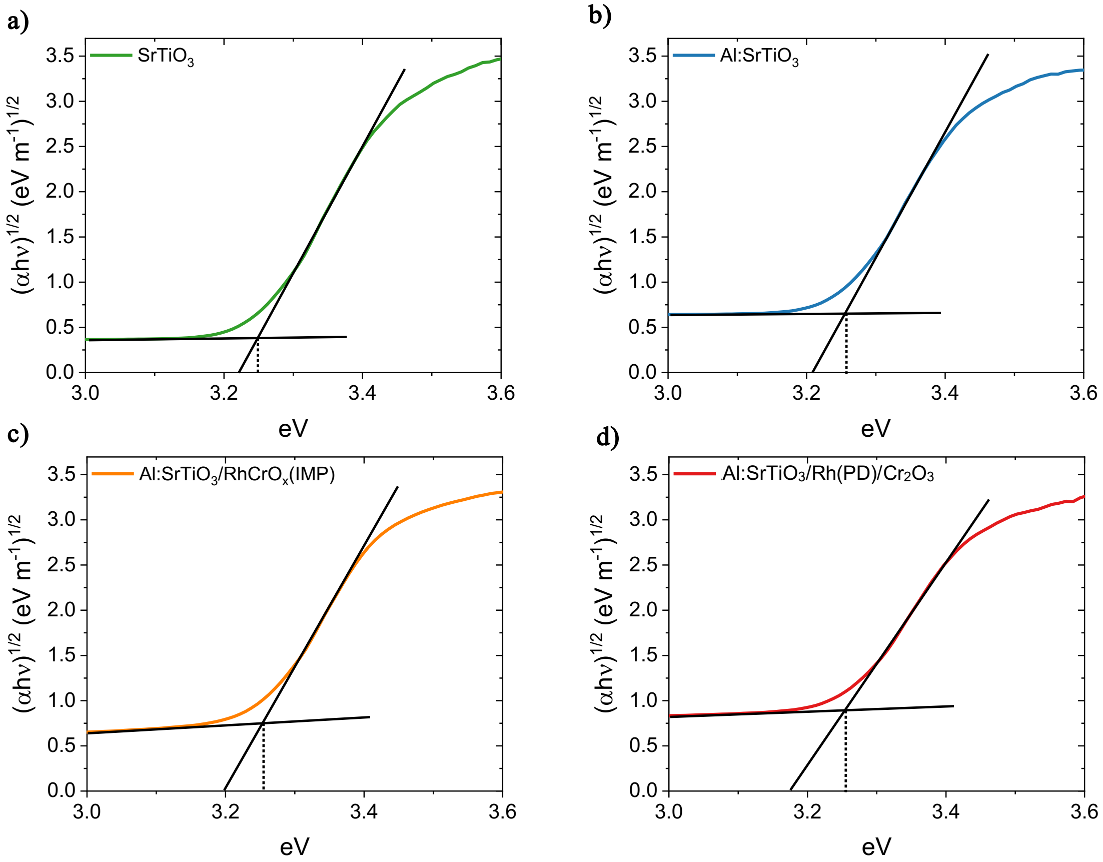

Figure S4: Tauc plots from optical data obtained by UV-Vis spectroscopy to obtain in indirect band gaps of a) $SrTiO_3$ (E_g = 3.25 eV), b) Al:$SrTiO_3$ (E_g = 3.25 eV), c) Al:$SrTiO_3$/$RhCrO_x$(IMP) (E_g = 3.25 eV) and d) Al:$SrTiO_3$/Rh(PD)/Cr_2O_3 (E_g = 3.25 eV).

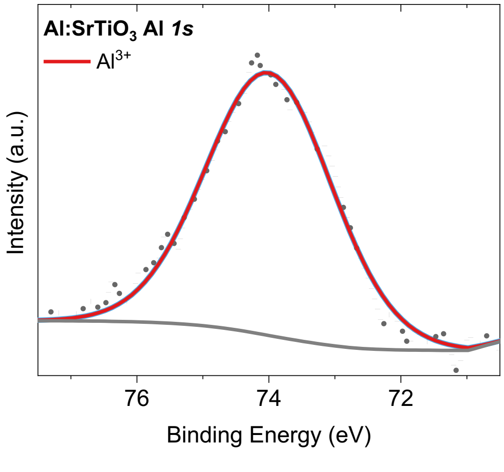

Figure S5: XPS Al 1s spectrum of Al:$SrTiO_3$ demonstrating the presence of Al^{3+} at the surface.

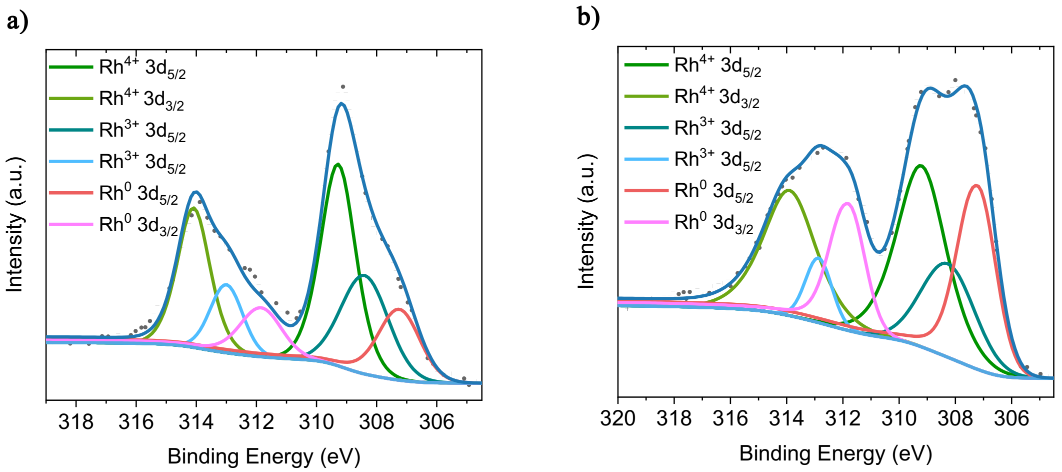

Figure S6: XPS Rh 3d spectra for a) $Al:SrTiO_3/RhCrO_x$(IMP) and b) $Al:SrTiO_3/Rh(PD)/Cr_2O_3$.

The higher contribution of metallic Rh in $Al:SrTiO_3/Rh(PD)/Cr_2O_3$ is expected due to the nature of the photodeposition method, whereby photogenerated electrons reduce the Rh source in the reagent mixture to metallic Rh. Consequently, Rh metal has a significant presence in the resulting Rh-Cr-based co-catalyst composition. In contrast, the impregnation method is recognised to predominantly yield Rh oxide species.[3,7]

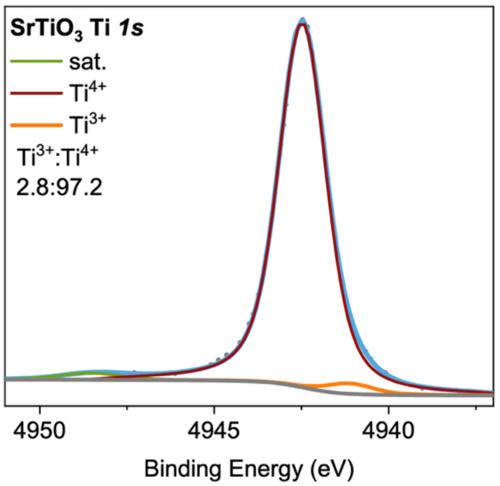

Figure S7: HAXPES Ti 1s core line spectrum of $SrTiO_3$ measured with hard X-Rays, showing a relative Ti^{3+} concentration of 2.8%.

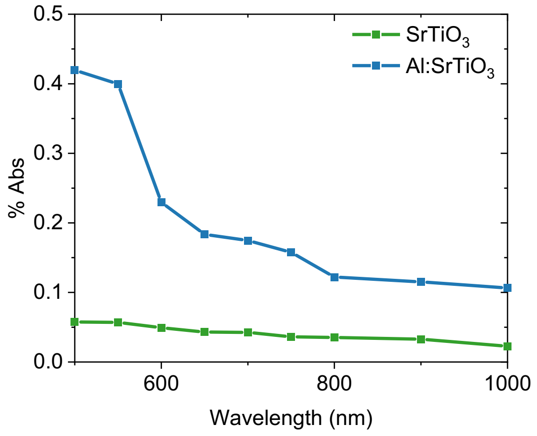

Figure S8: A comparison of the spectra of the maximum PIAS signals in H_2O of $SrTiO_3$ and Al:$SrTiO_3$, using a 365 nm LED at an intensity equivalent to 1 sun illumination (~2.7 mW cm^{-2}).

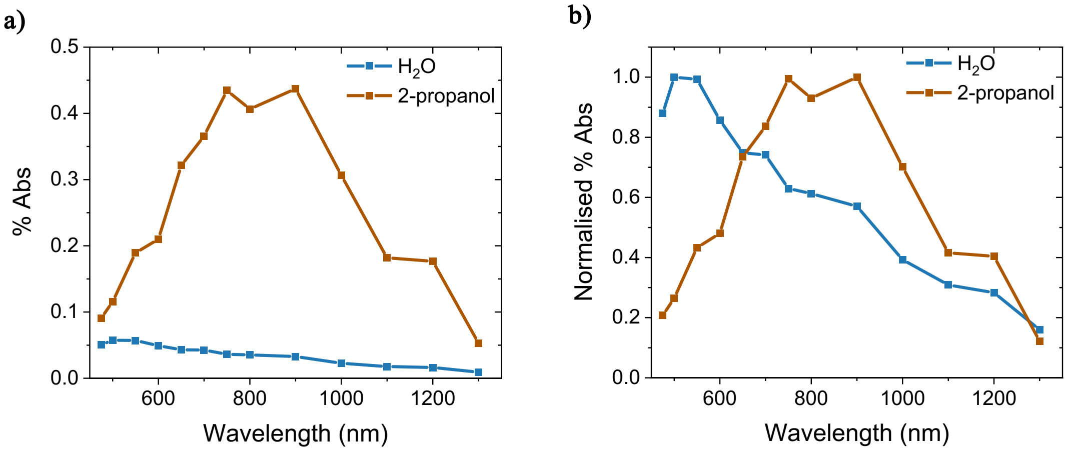

Figure S9: The impact of 2-propanol hole scavenger on the PIAS spectra of $SrTiO_3$ using a 365 nm LED at an intensity equivalent to 1 sun illumination, a) as measured, and b) normalised. In hole scavenger, the increase and decrease in signal amplitude observed above and below 650 nm respectively results in the assignment of hole species to below 650 nm and electrons at higher wavelengths.

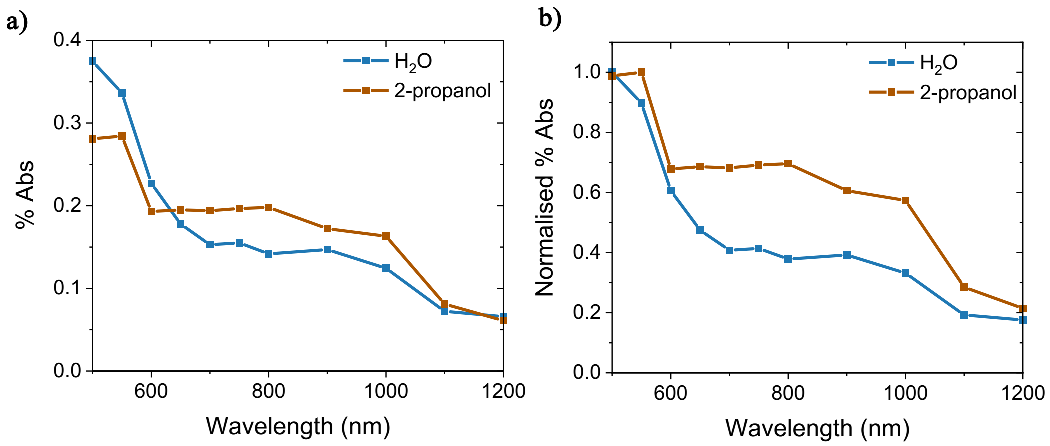

Figure S10: The impact of 2-propanol hole scavengers on the PIAS spectra of Al:$SrTiO_3$ using a 365 nm LED at an intensity equivalent to 1 sun illumination, a) as measured, and b) normalised.

The impact of hole scavenger is less dramatic than in $SrTiO_3$. However, an increase and decrease in signal amplitude above and below 650 nm respectively is still observed, to result in the same assignment of hole species to below 650 nm and electrons at higher wavelengths.

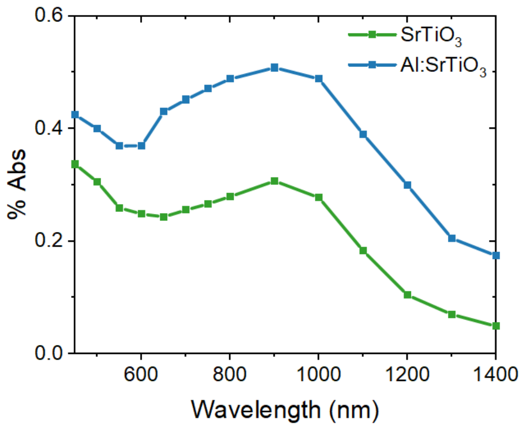

Figure S11: A comparison of the spectra of the DRTS signals at 10 μs in H_2O, of $SrTiO_3$ and Al:$SrTiO_3$ using a 365 nm LED at an intensity equivalent to 1 sun illumination, as measured to compare the signal intensities.

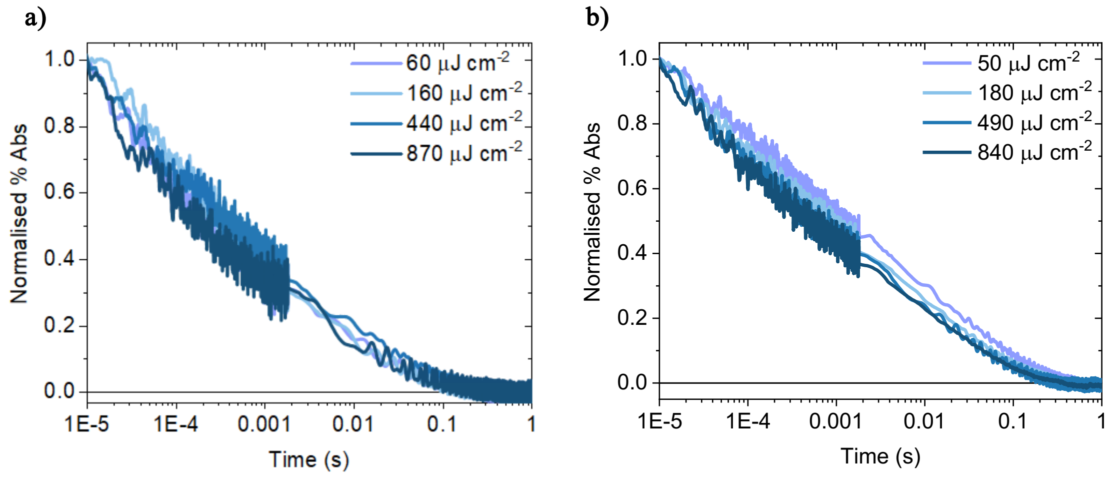

Figure S12: Normalised intensity dependence of DRTS kinetics probed at 500 nm in H_2O, for a) $SrTiO_3$, and b) Al:$SrTiO_3$. Measured using a 355 nm laser excitation at varying intensities.

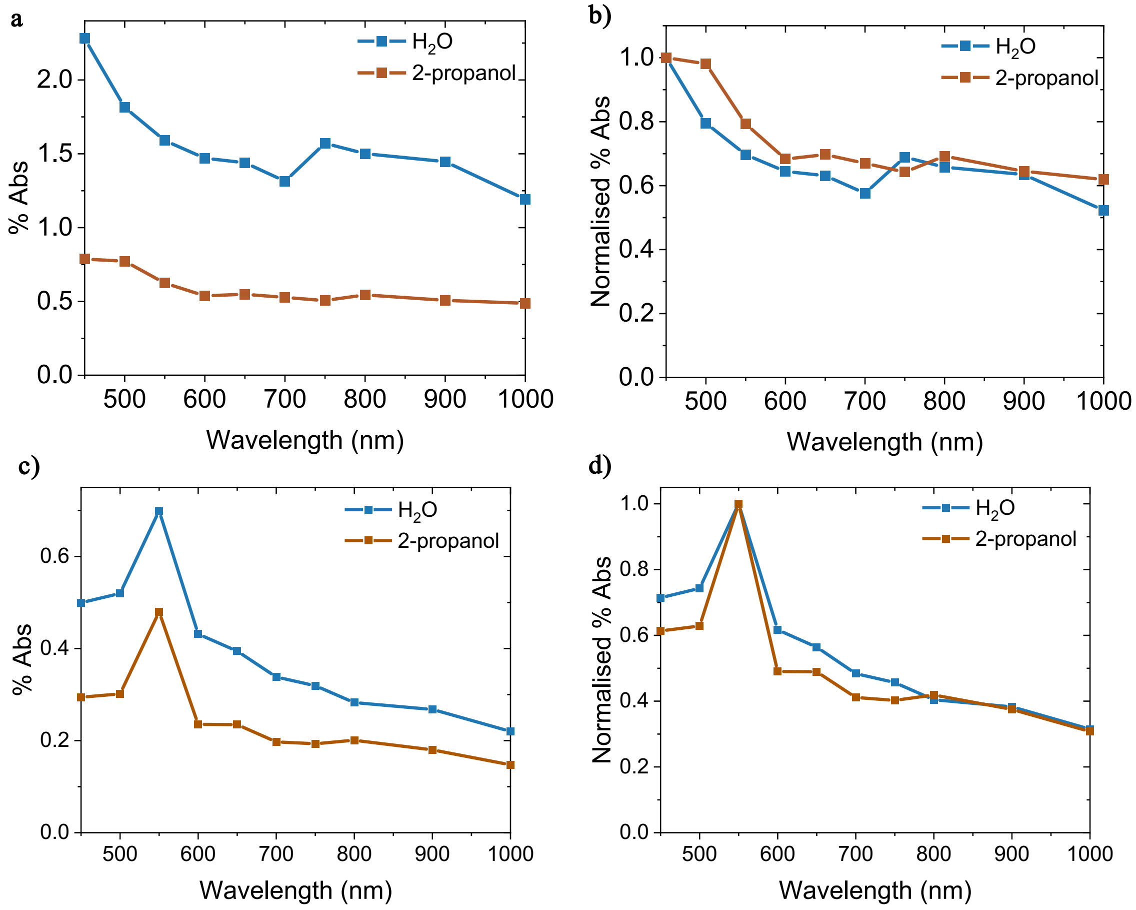

Figure S13: Effect of 2-propanol hole scavenger on the PIAS spectra of Al:$SrTiO_3$/$RhCrO_x$(IMP) a) as-measured and b) normalised, and on Al:$SrTiO_3$/Rh(PD)/Cr_2O_3 c) as-measured and d) normalised. All measured using a 365 nm LED at an intensity equivalent to 1 sun illumination.

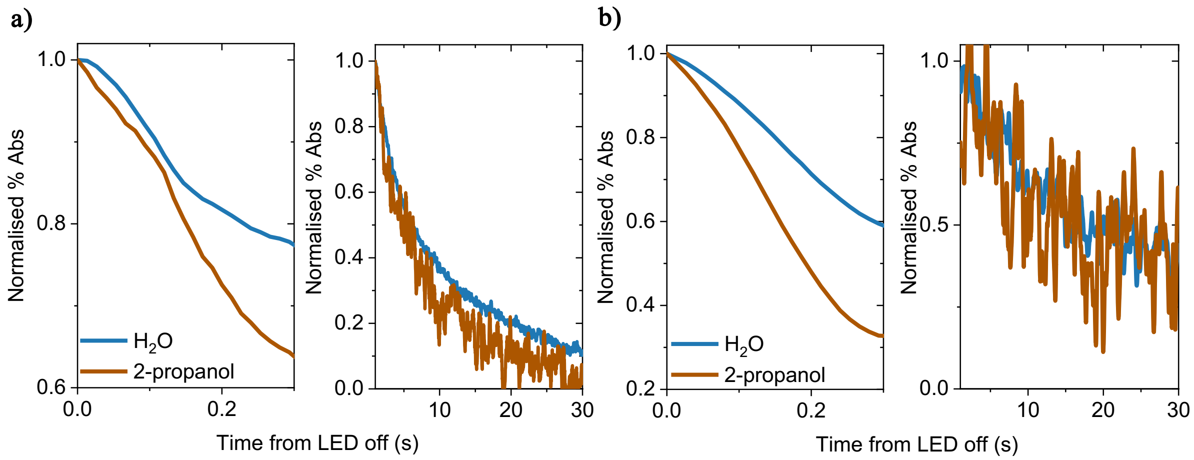

Figure S14: Comparison of the normalised fast (LHS) and slow (RHS) phase PIAS kinetics in H_2O and 2-propanol, probed at 500 nm, for a) Al:$SrTiO_3$/$RhCrO_x$(IMP), and b) Al:$SrTiO_3$/Rh(PD)/Cr_2O_3. Measured using a 365 nm LED at an intensity equivalent to 1 sun illumination.

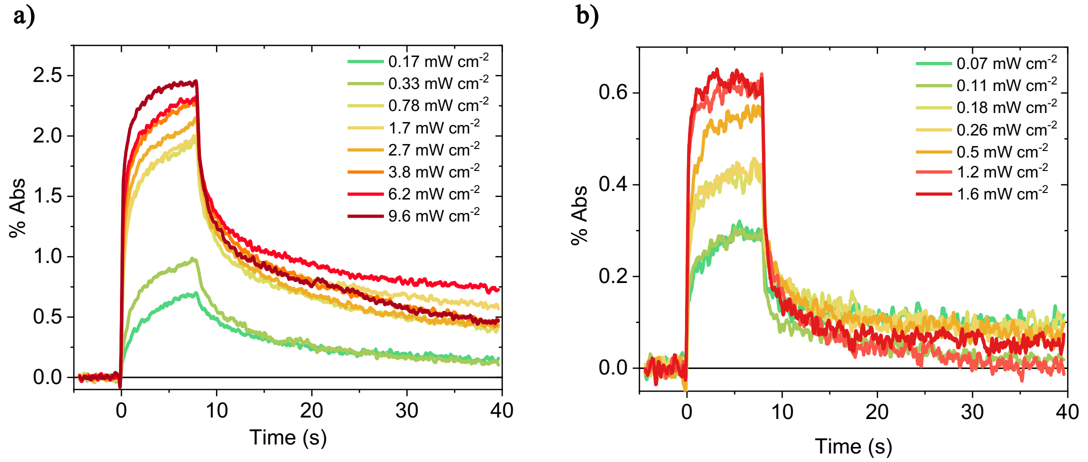

Figure S15: PIAS dependence on excitation intensity probed at 500 nm in H_2O for a) Al:$SrTiO_3$/RhCrOx(IMP), and b) Al:$SrTiO_3$/Rh(PD)/Cr_2O_3. Measured using a 365 nm LED at varying intensities, noting that the range of excitation intensities did not extend as high for Al:$SrTiO_3$/Rh(PD)/Cr_2O_3 due to intense bubble formation from Al:$SrTiO_3$/Rh(PD)/Cr_2O_3 interfering too significantly with spectroscopic measurements under higher intensities.

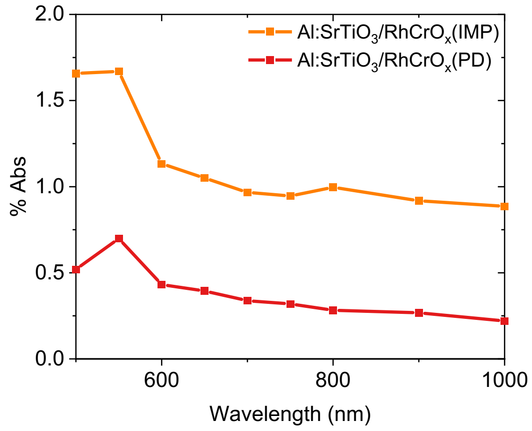

Figure S16: Comparison of the PIAS spectra of Al:$SrTiO_3$/$RhCrO_x$(IMP) and Al:$SrTiO_3$/Rh(PD)/Cr_2O_3, measured at 60% of 1 sun illumination intensity (~1.6 mW cm^{-2}) using a 365 nm LED. These measurements were undertaken at 60% sun illumination as intense bubble formation from Al:$SrTiO_3$/Rh(PD)/Cr_2O_3 interfered too significantly with spectroscopic measurements under 1 sun illumination.

a)

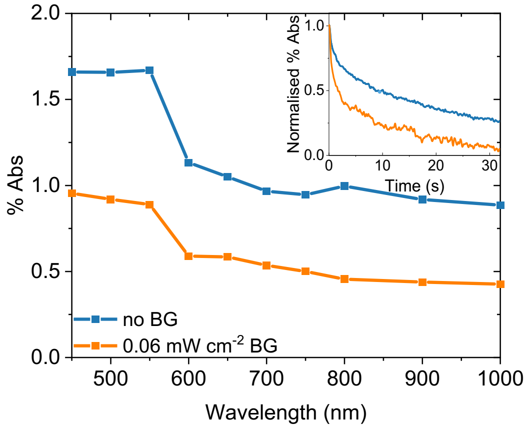

b)

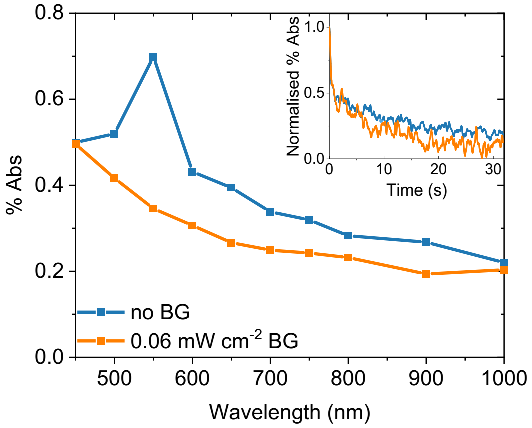

Figure S17: Comparison of the effect of background illumination on the PIAS spectra and decay kinetics at 500 nm (inset), of a) Al:$SrTiO_3$/$RhCrO_x$(IMP), and b) Al:$SrTiO_3$/Rh(PD)/Cr_2O_3. Measured using a 365 nm LED at an intensity equivalent to 60% of 1 sun illumination, with background illumination from an additional 365 nm LED.

a)

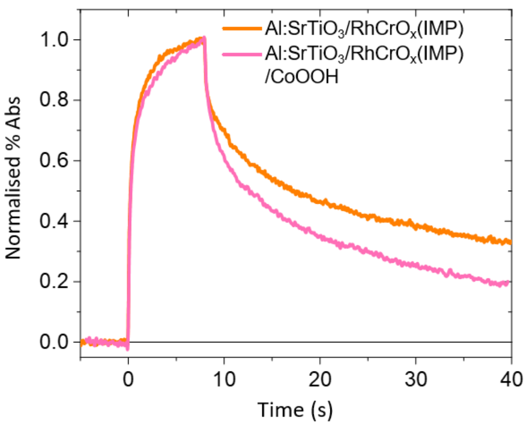

b)

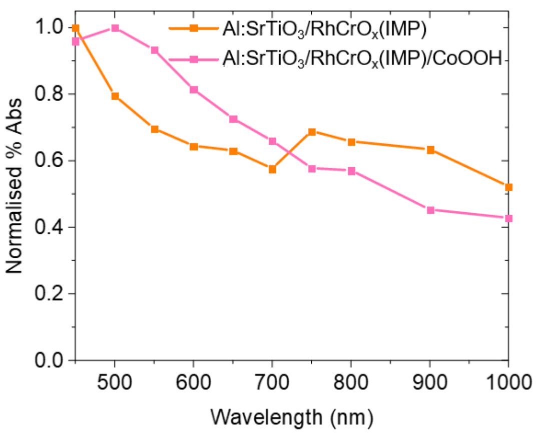

Figure S18: Comparison of Al:$SrTiO_3$/$RhCrO_x$(IMP) and Al:$SrTiO_3$/$RhCrO_x$(IMP)/CoOOH measured by PIAS in H_2O using a 365 nm LED at an intensity equivalent to 1 sun illumination, a) as the PIAS kinetics probed at 500 nm, and b) the PIAS spectrum.

References

1 Y. Ham, T. Hisatomi, Y. Goto, Y. Moriya, Y. Sakata, A. Yamakata, J. Kubota and K. Domen, *J. Mater. Chem. A*, 2016, **4**, 3027–3033.

2 T. Takata, J. Jiang, Y. Sakata, M. Nakabayashi, N. Shibata, V. Nandal, K. Seki, T. Hisatomi and K. Domen, *Nature*, 2020, **581**, 411–414.

3 K. Maeda, K. Teramura, D. Lu, T. Takata, N. Saito, Y. Inoue and K. Domen, *J. Phys. Chem. B*, 2006, **110**, 13753–13758.

4 T. Minegishi, H. Nishiyama, T. Hisatomi, M. Yoshida, H. Lyu, M. Katayama, K. Domen, Y. Goto, K. Asakura, Y. Sakata, T. Yamada, T. Higashi and T. Takata, *Chem. Sci.*, 2019, **10**, 3196–3201.

5 Y. Goto, T. Hisatomi, Q. Wang, T. Higashi, K. Ishikiriyama, T. Maeda, Y. Sakata, S. Okunaka, H. Tokudome, M. Katayama, S. Akiyama, H. Nishiyama, Y. Inoue, T. Takewaki, T. Setoyama, T. Minegishi, T. Takata, T. Yamada and K. Domen, *Joule*, 2018, **2**, 509–520.

6 S. Tanuma, H. Shinotsuka, C. J. Powell and D. R. Penn, *Surf. Interface Anal.*, 1993, **21**, 165–173.

7 K. Maeda, K. Teramura, H. Masuda, T. Takata, N. Saito, Y. Inoue and K. Domen, *J. Phys. Chem. B*, 2006, **110**, 13107–13112.

8 S. Selim, E. Pastor, M. García-Tecedor, M. R. Morris, L. Francàs, M. Sachs, B. Moss, S. Corby, C. A. Mesa, S. Gimenez, A. Kafizas, A. A. Bakulin and J. R. Durrant, *J. Am. Chem. Soc.*, 2019, **141**, 18791–18798.

9 S. Corby, L. Francás, A. Kafizas, J. R. Durrant, L. Francàs, A. Kafizas and J. R. Durrant, *Chem. Sci.*, 2020, **11**, 2907–2914.
